# An international overview of child protection services to protect unborn children from significant harm

**DOI:** 10.1080/09649069.2025.2530879

**Published:** 2025-07-21

**Authors:** Annick Zijlstra, Mariëlle R. Bruning, Maroesjka van Nieuwenhuijzen, Bram O. de Castro

**Affiliations:** a Research Institute Child Development and Education, University of Amsterdam, Amsterdam, The Netherlands; b Department of Child Law, Leiden Law University, Leiden, The Netherlands; c Expect Jeugd, Partners voor Jeugd, Amsterdam, The Netherlands

**Keywords:** Pre-birth child protection, significant harm, unborn child, legal comparative analysis

## Abstract

This paper presents a comparative analysis of the availability of pre-birth protection orders in cases of imminent harm to the unborn child due to high-risk parenting in the Netherlands and other Western countries. Using a standardised questionnaire sent to legal experts in 14 countries, the study evaluates the legal frameworks for such orders. Findings indicate that in most countries, pre-birth protection orders are unavailable, though some offer voluntary pre-birth protection proceedings or only prenatal care and support. Pre-birth protection orders are legally available in New Zealand and Norway. However, unlike the Netherlands, these countries have specific legislation allowing such orders, and pre-birth protection orders are rarely used. The study highlights the ethical and legal challenges of mandatory protection measures, emphasising the need for a balanced approach that respects the rights of both the mother and the unborn child. Recommendations include improving the accessibility and quality of voluntary care, and if pre-birth protection orders are available, this needs an explicit legal basis since this implies an infringement of the fundamental rights of women. This research provides a comparative legal analysis, underscoring the necessity for ongoing evaluation of these measures’ effectiveness and ethical implications.

All parents aim to bring their children into the world in a safe, loving environment and as healthy as possible. However, some pregnant mothers^1^ and their partners fail to protect their foetus and child from significant harm. Studies show that specific maternal behaviour harms the not-yet-born child and that this harm can persist into later life. For example, consuming alcohol during pregnancy, depending on the amount, duration, diet, concurrent substance use and genetics of the mother, can significantly increase the risk of foetal alcohol syndrome (Popova *et al*. 2023). Foetal alcohol syndrome is characterised by neurodevelopmental disorders with or without facial dysmorphology, congenital malformations and poor growth, with lifelong consequences (e.g. Blanck-Lubarsch *et al*. 2020, Popova *et al*. 2023). But also exposure to stress, smoking, or malnutrition during pregnancy increases the risk of premature birth, low birth weight, and long-term health problems, such as cardiovascular disease, diabetes and cancer (Barker 2007, Wadhwa *et al*. 2009). Additionally, there is a greater likelihood of social, mental and psychological problems, such as behavioural problems, learning difficulties and addictions (Norman *et al*. 2012). These disadvantages can continue throughout life and even into the next generation (Black *et al*. 2017, Richter *et al*. 2017). Moreover, the father’s behaviour can also harm the foetus. For example, abusive behaviour by the father towards the pregnant woman can increase her stress levels and alter foetal development (Glover and Capron 2017). In this paper, we refer to these harmful behaviours as ‘high-risk parenting’, which includes neglect, abuse, maltreatment, or any risk of significant harm to the child’s development.

Considering the long-lasting adverse effects of high-risk parenting, preventing harm to unborn children is critical for medical, economic, psychological, educational and biological reasons (World Health Organization, United Nations Children’s Fund and World Bank Group 2018). This necessity is underscored by the agendas of the WHO, UNICEF and the World Bank Group focussing on preventing harm to unborn children through early childhood parenting investments. They launched the Nurturing Care Framework in 2018, describing effective policies and services to support parents and caregivers in nurturing care (World Health Organization, United Nations Children’s Fund and World Bank Group 2018). Consequently, the number of low- and middle-income countries with a national policy for early childhood development has increased by 48% (World Health Organization and the United Nations Children’s Fund, 2023).

Such policies place the prevention of harm to unborn children within the context of prenatal care, considered a continuum of care from voluntary universal care, such as antenatal check-ups, at one end, and mandatory care, such as child protection or the forced hospitalisation of the pregnant woman, at the other (Hermanns 2008). Ideally, high-risk parenting is prevented by scaffolding vulnerable expectant parents through voluntary care. Preventive efforts have been shown to be feasible and effective for many families (e.g. Nurse-Family Partnership as described in Mejdoubi *et al*. 2015). However, in some families, high-risk parenting is hard to prevent without some form of mandatory child protection order. This paper focusses on whether and how mandatory pre-birth protection orders are organised internationally.

## The convention on the rights of the child

From an international children’s rights perspective, several standards can be deciphered. The Convention on the Rights of the Child (CRC) 1989 grants specific inherent rights to all children under 18 (*Convention on the Rights of the Child 1989*, Article 1), regardless of their geographic location (*Convention on the Rights of the Child 1989*, Article 2). While certain CRC articles and General Comments provide guidance on preventing harm to unborn children, they do not explicitly reference them. It was originally proposed to refer in Article 1 to ‘every human being from the moment of his birth’, but this original proposal led to considerable debate and these words were deleted from Article 1 (Detrick 1999).

However, the Preamble of the *Convention on the Rights of the Child (1989*) emphasises the need for special safeguards and care for children before and after birth, stating, ‘The child, because of his physical and mental immaturity, needs special safeguards and care, including appropriate legal protection, *before* as well as after birth’. However, since the Preamble is not legally binding, ratifying countries are not obligated to protect unborn children from significant harm (*CRC 1989*). When drafting the CRC, a prolonged discussion took place on the words ‘before as well as after birth’ that was also mentioned in the 1959 UN Declaration of the Rights of the Child. There was no consensus of a definition of the start of life in the CRC. Some delegations supported protection of the unborn child, but other delegations opposed these words because an unborn child was not literally a person whose rights could already be protected. The wording of the Preamble was a compromise; States Parties can adopt whatever position they wish, for the purposes of their own domestic law, on the issue of the right to life of the foetus or unborn child (Detrick 1999). Consequently, the enforcement of pre-birth child protection can vary between countries. The absence of a definition in the CRC does not preclude states legislating to protect unborn children but nor does it require them to do so or mean that ‘child’ must include ‘unborn child’. A woman’s right to choose to terminate a pregnancy and the availability of abortion time limits are actions which can be taken to protect the unborn child.

According to Article 31.1 of the Vienna Convention on the Law of Treaties 1969, a treaty should be interpreted in (a) good faith, (b) by the ordinary meaning of the terms of the treaty, (c) in light of the treaty’s context; and (d) in light of the treaty’s object and purpose (for further explanation see Broughton 2010). In this context, some argue that unborn children should also be entitled to rights without discrimination (e.g. Broughton 2010).

Relevant to preventing significant harm to unborn children is Article 19 of the CRC 1989, which states that children shall be protected from all forms of violence, with parents having primary responsibility for protecting the child and the State enabling them to provide a safe environment. In cases of serious concerns regarding the child’s safety, the government must intervene, preferably voluntarily, but with a child protection order if necessary. General Comment No. 13 (UN Committee on the Rights of the Child 2013) outlines the various types of violence addressed by Article 19, though it does not include violence during pregnancy.

Article 3 of the CRC 1989 states that the child’s best interests should be a primary consideration in all matters affecting children, including child protection orders. General Comment No. 14 (UN Committee on the Rights of the Child 2013) of the CRC elaborates on this. It does not explicitly define what is in the child’s best interests, but it provides an overview of reflections and considerations that should be taken into account when assessing what is in the child’s best interests.

Other relevant articles include Article 6 of the CRC 1989, which protects the child’s right to life and development, and Article 24 of the CRC 1989, which grants the right to the highest attainable standard of health, including adequate pre- and post-natal health care. General Comment No. 15 (2003) emphasises the health of the unborn child. Finally, there is also a General Comment that relates to all articles of the CRC, which emphasises that children’s rights also apply to very young children (zero to eight years) and that state parties should protect them (General Comment No. 7, CRC 2005).

## The Dutch system

In the Netherlands, it is a common practice that child protection orders are issued for unborn children. For example, 270 unborn children were subjected to child protection services in 2014 (Raad voor Strafrechttoepassing en Jeugdbescherming, 2015). Pre-birth protection orders have been issued in cases of maternal substance abuse, domestic violence and avoidance of (prenatal) care (e.g. [2018] ECLI:NL:RBNNE:2023:3217; [2020] ECLI:NL:RBLIM:2020:10620). The Dutch Civil Code 2021 allows (provisional) child protection orders when the child’s safety and well-being are at risk, and voluntary care is insufficiently accepted by the parents (2021, Article 255). However, the legislation is not explicit about the application of child protection orders for unborn children because the definition of ‘child’ is unclear. The Dutch legal system upholds the *infans-conceptus rule*, considering a child a legal person from conception if this is in their interests (2021, Article 2). However, the *infans-conceptus rule* is concerned explicitly with property law matters, not civil rights protection.

Moreover, the judiciary in the Netherlands is divided on applying the *infans-conceptus rule* for unborn children at risk of significant harm. Some judges consider that the application of this rule depends on the viability threshold of 24 weeks, which is related to the Termination of Pregnancy Law 1984 (e.g. [2021] ECLI:NL:RBDOR:2012:BV6246). Yet significant harm can occur early in pregnancy, as vital organs develop during this period and later damage cannot be prevented or healed (Moore *et al*. 2017). Consequently, there was a recent ruling where an unborn child was subjected to child protection services around the 16^th^/17^th^ week of pregnancy due to seriousness of the mother’s risky behaviour ([2020] ECLI:NL:RBDHA:2020:12532). This highlights the ambiguity in Dutch legislation and jurisprudence regarding pre-birth protection orders, not only as regards the possibility of imposing a pre-birth protection order per se but also as regards whether a pre-birth protection order can be imposed before the 24th week of pregnancy.

This evolving and, at the same time, ambiguous legal and policy landscape raises the question of whether and how child protection orders currently exist and are used in different countries to protect unborn children from significant harm. This paper presents an initial comparative analysis of the availability of mandatory pre-birth protection orders in Western countries.

### The current study

This study examines whether and how pre-birth protection orders to protect unborn children from significant harm due to high-risk parenting are legally organised in Western countries. This is an initial step in understanding and comparing how countries address this vital issue.

## Method

To examine whether, and how, pre-birth protection orders are organised internationally, we approached members of the International Society of Family Law from Western countries, including Ireland, the United Kingdom, Germany, Belgium, France, Austria, Denmark, Finland, Sweden, Norway, Australia, Canada, New Zealand, the United States and South Africa.

We utilised a questionnaire, asking: (1) can unborn children be legally protected by national laws and regulations in your country; (2) if so, under which laws and regulations (please copy the legal text); (3) how are these laws and regulations implemented; (4) how do these laws and regulations relate to the rights of the pregnant woman; (5) and – if they exist – how often are pre-birth protection orders imposed yearly. The questionnaire was sent to 30 members from 15 different countries. Referrals led to an additional 16 experts receiving the questionnaire. Upon receiving a response from a country, other respondents from that country were notified that their response was no longer required but still appreciated. Non-respondents were sent a reminder one week before the deadline of 1 March 2024.

Seventeen questionnaires were completed, providing information on the legal availability of pre-birth protection orders in 14 different countries. A caveat to note is that while our questionnaire did not specifically address voluntary measures, any information provided about such measures by respondents was considered a valuable by-product of the data collected. An internet search was conducted to verify and complete the information on pre-birth protection orders in each country. A summary was then written for each country and reviewed by at least the respondent and, if possible, a second person introduced by the respondent (9 out of 14 countries had a second review). Finland did not respond and was excluded. The response from the United States was excluded due to the state-level determination of pre-birth protection orders; the number of states is too large to summarise in one paper. The final countries (12) included were Ireland, the United Kingdom, Germany, Belgium, France, Denmark, Sweden, Norway, Australia, Canada, New Zealand and South Africa.

## Results

Table 1 provides an overview of the availability of pre-birth protection orders to protect unborn children from significant harm due to high-risk parenting in Western countries. Table 1 shows that most countries do not have pre-birth protection orders. These countries can be divided into two groups: those where no child protection orders are available during pregnancy, with only voluntary care and support from non-child protection organisations, and those where *mandatory* pre-birth protection orders are unavailable but *voluntary* pre-birth protection proceedings through child protection services are offered. In the latter group, families come under the provisions of child protection services, which may increase the likelihood of child protection involvement after birth. Only two other countries, aside from the Netherlands, have legislation that allows for mandatory pre-birth protection orders in cases of significant harm to the unborn child due to high-risk parenting. In this section, we describe for each country whether and how pre-birth protection orders are legally organised and provide background information on the legislative development.

**Table 1.** Overview of the availability of imposing pre-birth protection orders.

Country	Pre-birth protection order available	Act	Figures
Belgium	No		–
France	No		–
Sweden	No		–
Canada	No		–
South Africa	No		–
Denmark	No		–
Ireland	No, but voluntary pre-birth meetings are available.		Unknown
United Kingdom	No, but voluntary pre-birth meetings are available.		Unknown
Germany	No, but voluntary pre-birth meetings are available.		Unknown
Australia	No, but voluntary pre-birth investigations and meetings are available in New South Wales, Western Australia, Queensland and Tasmania. Prenatal reports are common in all states.		1529 substantiated reports in 2018–2019
New Zealand	Yes	*Oranga Tamaraki Act (1989)*	It has only happened twice since the existence
Norway	Yes	*Barnevernsloven (2024)*	20–30 per year
Netherlands	Yes	Case law is based on the interpretation of 2021, Articles 255 and 2.	270 unborn children in 2014

### Countries with only voluntary care and support

#### Belgium

Under current laws and policies, there is no specific legal provision for pre-birth protection orders to protect the unborn child^1^^1^The use of the word ‘child’ cannot be considered normative. Belgian researchers often use the more neutral term ‘unborn human life’ (Personal communication, 26 Jan 2024). in Belgium. Child protection only can be implemented after a child is born. If there are concerns about the welfare of an unborn child, the regional authorities can take preventive measures, albeit quite limited, including providing support and assistance to the expectant parents with their consent. There is currently no explicit legal possibility in Belgium to take preventive action without the consent of the expectant parents. Indirectly, measures such as the psychiatric commitment of pregnant women are sometimes applied.

However, developments seem to be underway that will make it possible to protect the child during pregnancy through a mandatory child protection order. As seen in De Mulder ((2022), several regional legal proposals (The Coalition Agreement for 2019-2024 (Government of Flanders, 2019); The Rousseau Decree 2021-2022 (Rousseau et al., 2021); the Concept Note Parys 2020-2021 (Parys et al. 2021)) describe that mandatory care for pregnant women will be developed by working with a pre-birth child protection order, based on the Dutch system. The Concept Note Parys 2020-2021 (Parys et al. 2021) is limited to pregnant women with addiction problems, whereas The Rousseau Decree 2021-2022 (Rousseau et al. 2021) is intended to cover situations where there is a ‘pregnancy of concern’, i.e. a pregnancy in which the interests and development of the unborn child are seriously endangered or threatened, thus necessitating judicial child protection measures (Article 2.7 of the proposal). The proposal reads that from the 13th week after conception, unborn children can be placed under the care of a family guardian, who will supervise the expectant mother and try to influence her “lifestyle” in a positive way. There are different opinions and criticisms about whether this will be an additional measure in the ‘Decreet Integrale Jeugdhulp’ (in the Integrated Youth Assistance Decree that applies explicitly to unborn children) or an extension of the current child protection system (see The Rousseau Decree 2020-2021 (Rousseau et al., 2021, p.15-16); and Concept Note Schryvers 2020-2021 (Schryvers et al., 2020, p.13)). Some see the first as preferable, as some existing measures could also be eligible for application during pregnancy, such as providing parents or parenting supervisors with an educational guideline (De Mulder 2022). As yet these are still proposals, and currently there is no possibility of a mandatory child protection order during pregnancy.

### France

In France, children cannot be subjected to child protection services before birth (Guedeney and Guedeney 2008). In case of concerns about the unborn child’s safety and development, France offers a wide range of free preventive care during pregnancy, regulated by the Agency for the Protection of Mothers and Children (Protection Maternelle et Infantile, PMI). The PMI consists of more than 5,100 centres across the country offering advice on contraception, access to social services and tailor-made solutions in PMI offices or at home, provided by professionals from all disciplines.

In France, a movement emphasises the importance of the first 1,000 days (Ministère du Travail de la Santé et des Solidarités 2020). From 2021, the government has set itself five priorities to encourage the creation of an environment conducive to the development of every child: (1) providing parents and relatives with simple, accessible and reliable information; (2) improving support for parents during pregnancy; (3) providing support in line with the needs and vulnerabilities of the parents, in particular for parents with learning disabilities; (4) encouraging parents to build a relationship with their child, by increasing the number of days of paternity leave; and (5) improving the quality of child-care for young children (Ministère du Travail de la Santé et des Solidarités, 2020). However, the use of mandatory measures to protect unborn children is not a focus of this movement.

### Sweden

In Sweden, there has been discussion about the possibility of pre-birth protection orders in the media (e.g. Dicksson 2018) and in court (e.g. [1987] SOU 1987:11), but currently the foetus is considered to be part of the pregnant woman’s body. Therefore, no protection orders are available in Sweden before the birth of a child.

When there are concerns about the unborn child during pregnancy, different organisations can work together to provide voluntary support and assistance to the pregnant woman and her family to protect the unborn child, e.g. specialist maternal care, support for recovery from addiction, preparation for parenthood, housing and basic needs. The provision of care during pregnancy is aimed at the woman’s need for support and is subject to her consent. To ensure effective cooperation, the law allows for the exchange of information between (maternal) health and social services without the permission of the pregnant woman herself if this is necessary for the protection of the unborn child (Public Access to Information and Secrecy Act, *Offentlighets- och sekretesslag 2009*:400, Chapter 26 Section 9 and Chapter 25 Section 12).

It is also possible for healthcare providers to report to social services (and vice versa) if they have concerns about the unborn child. However there is no obligation to report during pregnancy (*Offentlighets- och sekretesslag 2009*:400, Chapter 26 Section 9 and Chapter 25 Section 12). For example, social services might inform the hospital before delivery that the mother takes opioids, which is of great importance since the newborn may show severe signs of withdrawal (see, for example, [1987] SOU 1987:11, p. 82–83). When social services receive information about concerns about the unborn child, they can start a support programme with the expectant parents if they agree to the support. The interventions that can then be initiated are, therefore, voluntary. A duty to report exists only after the child is born; after reporting, social services can impose mandatory interventions under the Swedish Care of Young Persons Special Provisions Act (Lagen om vård av unga 1990, p. 52).

### Canada

Historically, Canadian provinces used birth alerts as a means to protect newborns when there were concerns about potential harm during gestation or if the mother had a history of involvement with child protection services. Birth alerts allowed social workers to request that hospital staff notify them as soon as the child was born, without the parents’ consent, often leading to immediate child protection orders and out-of-home placements (Sistovaris *et al*. 2021). As birth alerts were often based on stereotypes and assumptions they disproportionately affected Indigenous and other racialised mothers (Sistovaris *et al*. 2021), and were criticised as being illegal and unconstitutional due to the lack of parental consent (Carmant 2023). Therefore birth alerts have been discontinued across Canada (Hwang 2023).

In the current legal framework, Canadian law does not recognise unborn children as legal persons, and therefore, they cannot be protected by pre-birth protection orders during pregnancy (see for Court Cases on this topic: Abortion Rights Coalition of Canada 2024). The Canadian approach emphasises supporting pregnant women through voluntary prenatal care and early prevention programmes aimed at promoting healthy pregnancies and childbirth (Government of Canada 2020, Chapter 3). This includes educational programmes provided by community parenting and health centres. Research shows that such voluntary educational and supportive resources – covering areas like healthcare, housing, and income – significantly contribute to better outcomes for both the mother and the baby (McCalman *et al*. 2017). This approach prioritises respect for a woman’s autonomy while encouraging a healthy pregnancy and the birth of a healthy baby.

Court cases, such as Winnipeg Child and Family Services (Northwest Area) v DFG, [1997] 3 SCR 925, have reinforced the legal stance that unborn children do not possess rights under Canadian law. In this case, the court rejected a request for a pre-birth protection order to compel a pregnant, drug-dependent mother to enter treatment, ruling that no legal person existed to justify such an order. This decision underscores the broader legal principle that Canadian law does not recognise the unborn as legal persons with rights.

#### South Africa

In South Africa, there is no express provision made for the protection of the unborn from significant harm by employing a pre-birth protection order. The constitutional protection of persons begins at birth, and unborn children are not considered persons. This was held in Christian Lawyers Association of South Africa v The Minister of Health [1998] (4) SA 1113 (T). The Choice on Termination of Pregnancy Act 92 of 1996 further solidifies this position, under which abortion is legal without any reason provided to the 12th week of pregnancy and up to the 20th week of pregnancy if the social and economic circumstances of the pregnant woman are seriously affected; if the pregnancy poses a risk to the health of the pregnant woman; if the foetus would suffer a serious health risk; or if the pregnancy has resulted from rape or incest. Even after the 20th week of pregnancy, abortion is still legal if the life of the unborn child is at risk or there is a severe risk of birth defects. This underlines that the right to bodily integrity of the pregnant woman outweigh the right to protection of the unborn child. There has also been a case reaffirming that the unborn child is not considered a legal person. In this case of *S v Mashumpa and Another* (CC27/2007) [2007] ZAECHC 23; 2008 (1) SACR 126 (E) (11 May 2007), an unborn full-term baby was deliberately killed, but the judge acquitted the defendants.

It is unclear to the South Africa respondent what voluntary measures can be used when there are concerns about the child’s safety and development.

### Denmark

The Child’s Act (Barnets Lov 2024) contains several obligations for the municipality to protect the unborn child. First, it states that municipalities must supervise all children up to the age of 18, as well as expectant parents (Barnets Lov 2024, Article 9). In doing so, municipalities, citizens, and health and care professionals have to report in case of serious concerns about the unborn child (Barnets Lov 2024, Articles 132, 133, 135). Moreover, since 2007, it has been possible to offer voluntary social support to expectant parents if it is expected that the family and child will need social support after birth.

Under the Barnets Lov (2024), it is possible to decide during pregnancy, *without the consent of the future parents*, on the removal or adoption of the child immediately after birth. This is a possibility if there is a clear risk of serious harm to the health or development of the unborn child in the period after birth because of the future parents’ inability or lack of resources to provide appropriate care or treatment for the child (Barnets Lov 2024, Article 47(1)). However, no voluntary care is provided by child protection services during pregnancy, and only voluntary care and support are available. There are no recent figures on how often this measure has been used.

### Countries with voluntary pre-birth meetings

#### Ireland

In Ireland, there are no legal grounds for imposing pre-birth protection orders to protect an unborn child. However, voluntary pre-birth assessments do occur, which can often include recommendations that, if not followed, could lead to a basis for a care order upon birth (in line with the Guidance for the Safeguarding Process Prior To and Immediately After Birth of A Baby Where There May Be Risks of Significant Harm n.d..). This approach is an explicit choice of legislature that aims to respect the rights and choices of pregnant women within the context of Ireland’s reformed legal framework.

The repeal of Article 40.3 of the Irish Constitution in 2018 is essential in understanding why Ireland does not have a pre-birth child protection order and why there is strong opposition to such a provision. Article 40.3, often referred to as the Eighth Amendment had previously recognised the equal right to life of the mother and the unborn child. It effectively provided constitutional protection for the unborn, which had implications for various aspects of Irish law, including issues related to pregnancy and abortion. This provision was repealed following a national referendum on 25 May 2018. This allowed the Irish government to introduce more progressive legislation concerning women’s reproductive rights. For example, the Health (Regulation of Termination of Pregnancy) Act was introduced in 2018, which defines the circumstances under which abortion can be legally performed. The removal of Article 40.3, therefore, has direct implications for the possibility of imposing a pre-birth child protection order because the rights to life of the unborn child and the pregnant woman are no longer equal and imposing a child protection order during pregnancy could potentially conflict with the rights and choices of the pregnant woman. In post-repeal Ireland, the emphasis has been on upholding the rights and autonomy of pregnant women and ensuring their access to appropriate health care and support.

#### United Kingdom: England and Wales

The United Kingdom (UK) consists of four separate nations: England, Northern Ireland (this is a different jurisdiction to Ireland), Scotland, and Wales. However, the legal systems concerning child protection vary across these constituent countries. This paper focusses specifically on England and Wales, which share a common legal system related to child protection.

In both England and Wales, there is no provision for court proceedings or court orders before a child’s birth. This is related to the abortion law; abortion before 24 weeks is legal in all parts of the UK and in some specific cases beyond 24 weeks. Various attempts have been made in England to prevent legal abortions through injunctions or by making the child a ward of the court. In spite of these attempts, it has been held that a child is not a legal person and, therefore, legal action cannot be taken to intervene to protect a child before birth, see *Re F (in utero)* [1988] Fam 122.

Despite the lack of legal personhood for unborn children, child protection authorities in England and Wales can take action during pregnancy through child protection planning, although legal proceedings for child protection, supervision, or child removal can only take place after birth. This process, which is not a statutory one, but is guided by the *Working Together to Safeguard Children Guidance* (HM Government 2023), allows for the holding of a child protection conference, making a child protection plan and appointing a key worker. The guidance specifies that local protocols should address the specific needs of certain groups, including unborn children where there are concerns. Thus, child protection planning depends on local protocols, which may vary from area to area and significantly depend on resources.

Generally, the process unfolds as follows: individuals concerned about an unborn child’s welfare can contact a local authority, which will conduct an initial assessment. If this assessment indicates a risk of significant harm to the unborn child, an investigation can commence in the context of section 47 of the Children Act (1989). This investigation involves gathering information from various professionals, parents and family members to determine if the unborn child is currently suffering or is at risk of significant harm. If there is potential for significant harm, an initial child protection meeting may be held before birth. A child protection plan can be made, outlining how the child can be protected immediately after birth and into the future. Almost 900 unborn children had a child protection plan in England in 2013 (as described in Masson and Dickens 2015).

In the context of pre-birth child protection planning, it is also essential to mention the pre-proceedings system in England and Wales (Department Education 2014, Welsh Assembly Government 2014), which is a semi-formal process before care proceedings are initiated. The pre-proceedings process guides working with families on the edge of child protection services, even before birth (Masson and Dickens 2015). The framework outlines the steps that local authorities should take before starting child protection proceedings, provided the immediate safety and welfare of the child are not compromised. Parents are sent a letter entitled ‘How to avoid going to court’ - before proceedings, which sets out the concerns of children’s services and the changes they should make and invites them to a ‘pre-proceedings meeting’. The aim is to involve parents in working with the local authority and to avoid child protection proceedings, or where this is not possible, to reduce conflict (Masson and Dickens 2015). The pre-proceedings process is commonly used in child protection planning in situations with unborn children; 30% of pre-proceedings processes were in relation to unborn children (Masson and Dickens 2015).

### Germany

In Germany, the unborn child is protected against significant harm by various laws and measures, both criminal and civil (see for an overview Deutscher Bundestag 2020). This paper focusses explicitly on pre-birth protection orders under civil law. The extent to which civil protection measures apply to unborn children in Germany remains unclear. In Germany, Article 1666 of the German Civil Code (Bürgerliches Gesetzbuch 1774) provides for child protection measures to protect the child from significant harm. However, there are two perspectives on the interpretation of this Article as regards unborn children. The first perspective argues about whether protecting the unborn child under Article 1666 Bürgerliches Gesetzbuch (1774) also extends to the pregnancy period, i.e. the unborn child. The wording of the provision refers to the child as a legal person. According to Article 1 Bürgerliches Gesetzbuch (1774), legal personality begins with the completion of birth, just as parental custody starts with birth. In addition, the Bürgerliches Gesetzbuch (1774) contains explicit provisions for the protection and legal status of the unborn child; Article 1666 is not one of these explicit exceptions.

Contrary to this, another viewpoint suggests that the unborn child already enjoys the protection of Article 1666, as strict literal interpretation conflicts with constitutional provisions safeguarding unborn life. From this perspective, constitutional protections under Article 1(1) and Article 2(2) of the Constitution (Grundgesetz 1949) support a comprehensive interpretation of Article 1666, aligning with the Constitution’s principles and including the unborn child. Denying protection through family law’s central authorisation would thus contradict the State’s duty to protect. This is also seen in Article 6(2) of the Grundgesetz, which states that the state supervises parents’ care and upbringing rights. This so-called guardianship mandate specifies the state’s duties to protect the child’s fundamental rights and grants the state a subsidiary care and education mandate. The guardianship mandate also covers the pre-birth period if there are specific indications that the unborn child is in danger, for example, due to the pregnant woman’s drug addiction.

Regardless of a definitive decision on the aforementioned legal question, it is observed in practice that a guardian can be appointed during pregnancy (German Civil Code (Bürgerliches Gesetzbuch 1774), Article 1912 and Article 1774(2)), but their role can only commence after the child has been born. However, the guardian has no concrete power to influence the pregnant woman, which can only be exercised after birth (Deutscher Bundestag 2020).

### Australia

All Australian states (New South Wales, Queensland, South Australia, Tasmania, Victoria, Western Australia) do not consider the unborn child as a legal person. This limits mandatory child protection services to after a child is born (Wise *et al*. 2021). However, in all states of Australia, expectant parents can be reported to child protection services during pregnancy (Wise *et al*. 2023). This is referred to as ‘unborn child reports’ or ‘prenatal reports’ (O’Donnell *et al*. 2023). The aim of reporting to child protection services during pregnancy is to collaborate with the expectant parents to reduce risky behaviours that might affect the development and safety of the unborn child, to prevent out-of-home care after birth and to identify cases where out-of-home care is necessary after birth (Taplin 2017). However, the response to the reports varies by state.

Only in Tasmania it is mandatory to report when it is reasonable to believe that the unborn child will be suffering abuse or neglect, or may be killed by a person with whom the child will be living, or that the child needs medical treatment or intervention due the behaviour of the pregnant woman or another person (Children, Young Persons and Their Families Act 1997, Sections 3, 4 and 14). In other states, there is no reporting requirement during pregnancy, but reporting is possible. Although it is not required in New South Wales to report, once an unborn child report has been substantiated, mandatory reporters must report if the pregnant woman fails to attend antenatal services (Australian Institute of Family Studies 2020). The grounds for an unborn child report vary by state, ranging from factors such as teenage parenthood and homelessness to intellectual disability and substance abuse (for an overview, see Wise *et al*. 2023).

The responses to these reports differ across states. The mandatory reporting laws for each state define what must be reported; the legislative grounds define when it is acceptable for child protection services to intervene (Child Family Community Australia 2023). The states New South Wales, Western Australia, Queensland and Tasmania have specific provisions for investigating during pregnancy, stating in Queensland that there must be explicit consent from the pregnant woman to participate in the investigation, i.e. the investigation must not interfere with the rights and liberties of the pregnant woman (Queensland Government, n.d.). In Victoria, on the other hand, a report during pregnancy is not classified as a protective intervention report; thus, no investigation, substantiation, or intervention takes place (Victorian Health and Human Services 2021). Moreover, the Australian Capital Territory, Northern Territory and South Australia do not conduct prenatal investigations (O’Donnell *et al*. 2023; Wise *et al*. 2023). In the Northern Territory, the information from any report during pregnancy is used to determine whether a child protection intervention is needed after birth (Territory Families 2020).

When the outcome of the investigation is that child protection intervention is needed after birth, child protection services remain involved until birth in the states of Queensland and the Australian Capital Territory (Community Services 2018; Department of Child Safety, Youth and Women 2020 as described in Wise *et al*. 2023). In New South Wales the risk assessment gives input for the safety plan, which is made in collaboration with the expectant parents (Wise *et al*. 2023). Involvement of child protection during pregnancy aims to prevent risks after birth and to provide appropriate help. However, if parents avoid child protection involvement, a plan can be made in their absence to be implemented after birth. Child protection services can request a ‘high-risk birth alert’ from the hospital to inform child protection services when the birth is expected or when the child is born.

A study by O’Donnell and colleagues (2023)^3^ found that the rate of reports during pregnancy increased by 4% per year over seven years (2012–2013 to 2018–2019). In 2018–2019, the total number of reports during pregnancy in Australia was 24.4 per 1000 births, which equates to 5384 children. One out of three reports during pregnancy was substantiated in 2018–2019 (1529 children). Neglect was substantiated in 59% of cases, and emotional abuse in 21%.

### Countries with a pre-birth protection order

#### New Zealand

In New Zealand, the unborn child can be placed under the mandatory supervision of the Oranga Tamariki – the Ministry for Children, although this has only happened twice: In the Matter of Baby P [1995] NZFLR 577 (FC) and Re an Unborn Child [2003] 1 NZLR 115 (HC). Oranga Tamariki – Ministry for Children replaced the Government Agency for Child Protection Services in 2017 because the Government Agency was failing to meet the needs of children in need. Oranga Tamariki is child-centred and focusses on prevention and early intervention, i.e. during pregnancy (Burgess 2019).

Although rarely used, the Oranga Tamariki Act 1989 allows for a mandatory protection order to be issued before birth. This Act sets out the Ministry’s care and protection powers. The premise of the Act is that the welfare and best interests of the child or young person are paramount. A report of concern can be made under section 15 of the Act. A report of concern is ‘a report to the agency that a child or young person has been, or is likely to be, harmed, ill-treated, abused, neglected or deprived’. When a report is received, the Oranga Tamariki may investigate the report if necessary or desirable under Section 14 of the Act. However, it is at the Oranga Tamariki’s discretion whether this is necessary or desirable. The Oranga Tamariki will then decide whether to continue the investigation with a family group plan or refer the case to the court for further proceedings to protect the unborn child. Besides a (pre-birth) protection order under section 78 of the Act as referred to in this paper, other protective measures are available as described in Section 2. The court will only issue a protection measure if other measures, including a family group plan, have proved inadequate.

In 2017–2018, the Oranga Tamariki received 1,949 reports of situations of concern for the unborn child, where the unborn child was considered likely to be harmed, ill-treated, or abused. In 1,235 cases, the Oranga Tamariki offered further voluntary protection measures under Section 2 of the Act (Burgess 2019). Since its existence, only two mandatory pre-birth protection orders have been imposed.

### Norway

In Norway, as of 1 January 2024, under section 2–4 of the Child Welfare Act (*Barnevernsloven 2024*), the Child Welfare Service can open a child welfare case without the consent of the pregnant woman if a pregnant woman is admitted to an institution under Section 10–3 of the Health and Care Services Act (Lov om kommunale helse- og omsorgstjenester 2023). A pregnant woman can be admitted if there is a reason to believe that the woman is abusing intoxicants in such a way that the baby is likely to be born with a defect. She can be kept there for the entire duration of the pregnancy if other care measures cannot prevent harm to the child. The purpose of the detention is to limit the harm to the child, to provide the pregnant woman with adequate support for her abuse and to enable her to take care of her child. After every three months of detention, the municipalities reassess whether there are still grounds for detention. Although opening a child protection case during pregnancy has only been possible since 2024, it has been possible to refer a pregnant woman to an institute under section 10–3 of the Lov om kommunale helse- og omsorgstjenester. The latest figures indicate that this happens rarely; in 2016, 28 decisions were made under sections 10–3 and 35 temporary decisions ordered (Folkehelseinstituttet 2018). Therefore, the act seems more to be a pressure for voluntary cessation of drug use.

The child welfare service can also offer voluntary assistance and assess the need for measures after birth (*Barnevernsloven 2024*, Section 2–4). Moreover, since adopting the new Act, child protection services have been authorised to process personal data in cases involving unborn children (*Barnevernsloven 2024*, Section 13–6). However, this does not expand the responsibility of child protection services in situations involving unborn children. In the absence of a reason under Article 10–3, child protection services are still dependent on the consent of the expectant parents to open a case.

## Summary of findings

To summarise, compared to other countries, Dutch practices regarding mandatory pre-birth child protection orders appear to be quite unusual. Most reviewed countries do not have legislation allowing mandatory pre-birth protection orders. These countries fall into two groups: those offering only voluntary care and support without child protection services, and those with voluntary pre-birth protection proceedings available from child protection services. In this first group are countries that emphasise voluntary support programmes and education to promote healthy pregnancies and births from the perspective of pregnant women’s fundamental human rights, such as Canada and France, while others are considering the introduction of mandatory pre-birth protection orders, as in Belgium and Sweden. Further steps have already been taken in this direction in Denmark, where from 2024 it is possible to impose a child protection measure – in the form of out-of-home placement or adoption *after* birth – during pregnancy.

In the second group of countries where there are no mandatory pre-birth protection orders, *voluntary* pre-birth protection proceedings are available. This is the case in Ireland, the United Kingdom, Germany and Australia. In Ireland, voluntary pre-birth assessments can lead to post-birth care orders. In the United Kingdom and Australia, child protection proceedings during pregnancy require parental consent, but a mandatory protection measure can only be imposed after birth. Germany allows the appointment of a guardian during pregnancy, but their mandatory powers can only be exercised after birth. Thus, in these countries, assessments can be carried out during pregnancy with parental consent to determine whether a child protection order at birth is needed or to prevent child protection involvement at birth. However, this can still be perceived as coercion and not all parents experience this as a voluntary option.

Only New Zealand and Norway, aside from the Netherlands, have mandatory pre-birth protection orders. New Zealand uses them if other means are impractical. Norway’s orders address substance abuse. However, compared to The Netherlands, these countries rarely use mandatory orders, implying that these countries have almost always persuaded the parents, through voluntary care, to prevent harm to the unborn child. Furthermore, unlike in the Netherlands, these measures have an explicit legal basis that was decided upon by a democratic process.

Overall, all included countries navigate complex legal, ethical, and social considerations for protecting the unborn child, emphasising the need for comprehensive and nuanced approaches to prenatal care and child protection. The Netherlands stands out for frequently imposing pre-birth orders without a clear legal mandate.

## Conclusion & discussion

All parents aim for a safe and healthy childbirth, but some fail due to high-risk behaviours. Preventing harm to the unborn child ideally involves voluntary care and support, but mandatory orders might be needed in exceptional cases. International treaties do not impose explicit obligations on states to protect the unborn child, leaving it an ethical debate. In the Netherlands, the judiciary regularly applies mandatory pre-birth protection orders for unborn children by applying regular child protection orders, even though they were not explicitly introduced for unborn children by the legislator. This paper examined how this Dutch practice relates to other countries with an initial comparative analysis – resulting from a collaboration between legal and child development and education experts – of whether and how pre-birth protection orders exist in Western countries to protect unborn children from significant harm, based on a methodological survey of respondents.

It can be concluded that in most countries covered in this paper, pre-birth protection orders are unavailable, and where they exist, they result from careful consideration in the form of a democratic process, and political debate has been given to the reasons why pre-birth protection orders are available or not during pregnancy. For example, Ireland has explicitly chosen not to implement pre-birth protection orders in light of its legislation on women’s reproductive rights. In contrast, in the Netherlands, a specific legal threshold for a pre-birth protection order is lacking, no legislative process with political discussion has been followed, and decision-making is thus left to the judiciary.

Importantly, in the countries that do have mandatory pre-birth protection orders, these are rarely used because intensive tailored assistance on a voluntary base is preferred and accepted by parents. This contrasts markedly with the situation in the Netherlands, where children’s judges can impose pre-birth protection measures, and have applied them a few hundred times yearly. In this sense, the Netherlands differs considerably from all other countries in this analysis. These outcomes should motivate the Netherlands to be more reluctant to use regular child protection orders for unborn children without a specific legal basis, without political consensus, and without having tried improving (access to) services provided voluntarily.

In countries without mandatory pre-birth protection orders, a conscious decision has been made not to do so. However, this does not mean that there is no support available for expectant parents to prevent harm to the unborn child, either in the form of voluntary child protection proceedings or voluntary care and support. In some of these countries, child protection services attempt to persuade parents to accept support, while others rely solely on consent-based support due to the mothers’ fundamental rights. Studies show that voluntary care effectively prevents harm to the unborn child (e.g. McCalman *et al*. 2017), including in the Netherlands (e.g. Mejdoubi *et al*. 2015), though the high number of pre-birth protection orders there suggests underutilisation or inefficacy of voluntary measures, raising questions about balancing voluntary and mandatory approaches and the effectiveness of current support systems.

Although pre-birth protection orders are unavailable in most countries, it is striking that many countries have recently shifted their focus to the protection of unborn children. This growing attention may stem from the emerging focus in the social sciences on the negative outcomes for children exposed to significant harm during pregnancy (see Introduction). For example, countries like New Zealand have already identified and acted on opportunities to protect the unborn child through mandatory measures during pregnancy. Similar developments have taken place in Norway, with the recent reforms allowing for the imposition of mandatory child protection measures during pregnancy. Meanwhile, other countries such as Belgium and Germany are actively exploring their options, reflecting a wider international effort to address this issue even in the absence of formal pre-birth protection orders.

While this comparative analysis offers valuable insights into the varying approaches to pre-birth protection orders across different countries, several limitations must be acknowledged. First, when interpreting the results of this study, the continuously developing landscape in this area should be considered. The results focus on the period up to March 2024; in the meantime, new developments may have occurred. Secondly, a comprehensive overview of the content and the effectiveness of all the measures mentioned (pre-birth protection order, voluntary pre-birth protection proceedings, voluntary care and support) is lacking in this study. The limited use of mandatory pre-birth protection measures in New Zealand and Norway may suggest that these interventions are not effective. Follow-up research should focus on understanding why specific legal provisions fall into disuse and the implications of a shift in emphasis towards family support.

Additionally, each country’s cultural and socio-economic contexts significantly influence the effectiveness and acceptance of voluntary and mandatory child protection approaches. For example, the differences between collectivist parenting and individualistic parenting (Lansford *et al*. 2021), as well as austerity measures play a role in the availability and design of measures (Rajmil *et al*. 2020). Such influences were not included and examined in this paper. To this end, follow-up research should investigate the evaluation of these measures’ efficacy and impact on protecting unborn children to understand how these contextual factors impact the outcomes of pre-birth protection measures. Finally, the study does not account for all stakeholders’ perspectives, including parents, child protection workers and legal professionals, which could provide a more comprehensive understanding of the issues at hand.

Based on this research, the Dutch approach to imposing pre-birth protection orders is exceptional internationally, and this practice seems questionable to us. Pre-birth protection orders include an intervention in a pregnant woman’s life and the fundamental rights of both the mother and unborn child. Therefore, we question whether such decisions should be left to the judiciary. Relying on judges to determine if a regular child protection order can be applied in pre-birth situations, without an explicit legislative mandate, leads to legal uncertainty and potential inequality.

Therefore, we would like to make several recommendations. First, improve the accessibility and quality of voluntary care. The severe intrusion of a pre-birth protection order on a pregnant woman’s life and freedom of choice – whether or not to carry the pregnancy to term – raises questions about the necessity of mandatory intervention, given the effectiveness of voluntary measures. Moreover, as follows from Article 18(2) of the CRC 1989, states must support parents in exercising their parental responsibilities and evaluate the effectiveness of the support. From this perspective, voluntary support should remain the starting point, including general antenatal care and targeted support for high-risk parents, which can reduce high-risk parenthood and avoid coercive child protection measures. The government should evaluate the provision of support and its effectiveness.

However, voluntary support can still be perceived as coercive due to the fear of mandatory measures after birth (De Mulder 2022). Both voluntary care and support, and voluntary pre-birth protection proceedings often seem to be experienced as a disguised form of pressure. In our opinion, therefore, a measure is truly voluntary only if it does not threaten to become mandatory after birth.

From this follows our second recommendation: whether mandatory pre-birth protection orders are possible or not should be explicitly stated in the law, resulting from a democratic process with a public and political debate about state intervention in the private life of a pregnant mother. A legal basis for pre-birth protection orders for unborn children is necessary to uphold the fundamental human rights of pregnant women. Specific in the Netherlands, the 2015 Criminal Justice Council report entitled ‘Prenatal child protection and the role of the state’ (Raad voor Strafrechttoepassing 2015), called for more legal powers to protect the unborn child through a new pre-birth protection measure, as the current application of the child protection measure is not designed and tailored to the situation of the unborn child. Implementing mandatory pre-birth protection measures would necessitate that parents are fully informed about the potential consequences of their actions and behaviours (e.g. by your behaviour you are creating an unsafe situation for your child which may result in a child protection order being made). An illustrative example of this can be found in the pre-proceedings system in England and Wales.

Third, pre-birth protection orders often occur after the viability threshold, which in the Netherlands is 24 weeks. Literature shows severe damage can occur before 24 weeks Black *et al*. (2017); Richter *et al*. (2017). Early intervention could mitigate risks more effectively and potentially avert drastic measures later in pregnancy. This highlights a significant gap in the current legal framework prioritising foetal viability over developmental safety. Policies allowing for timely and proactive protection of the unborn child are needed.

We believe that, with the above recommendations, mandatory pre-birth protection can be prevented far more often than currently seems to be the case in the Netherlands (e.g. twice in two decades in New Zealand versus 270 times per year in the Netherlands) and can only be justified in exceptional cases, in which both parents are granted legal protection, and the unborn child is protected from significant harm. Ultimately, our message is that the Netherlands should take legislative action and should no longer accept mandatory child protection orders for unborn children by applying regular child protection orders based solely on judicial discretion.

This international comparison has shown that policymakers can learn from analysing law and practice in other countries. Individual countries’ policy options may have already been tried elsewhere, with effects that may be learned from. Moreover, a practice that may have grown historically and has become ‘common practice’ in a specific country (such as pre-birth protection orders for unborn children in the Netherlands) may turn out to be exceptional and in need of open debate when compared to practice in other countries. We hope this review may thus contribute to societal discussion on protecting unborn children and the roles of voluntarily providing high-quality support and possibly protecting orders therein.
